# The psychological impact of COVID-19 on healthcare work force in the Middle East region C-S study

**DOI:** 10.3389/fpsyt.2023.1112501

**Published:** 2023-03-03

**Authors:** Marwa Ahmed El-Gammal, Amira Elgendy, Petra Heidler, Tarek A. Owais, Nael Kamel Eltewacy, Nouran Hamza, the EARG group, Mohammed Amir Rais

**Affiliations:** DMD, Algiers Department of Dental Medicine, Algeria: Faculty of Medicine, Al-Azhar University, Egypt: Faculty of Pharmacy, Damanhour University, Egypt: Faculty of Pharmacy, Sohag University, Sohag, Egypt: Faculty of Pharmacy (Boys), Al-Azhar University, Assiut Branch, Egypt: Faculty of Pharmacy Mansoura University, Egypt: Faculty of Medicine and Health Sciences, An-Najah National University, Nablus, Palestine: Faculty of Medicine, Al-Quds University, Palestine: Faculty of Medicine of Tunis, University Tunis El Manar, Tunisia: Faculty of Medicine of Monastir, Tunisia: Faculty of Medicine Sfax, Sfax University Tunisia, Sfax, Tunisia: Faculty of Medicine, Aleppo University, Syria: College of Medical Technology, Department of Public Health, Derna, Libya: Radiologist Almogarief Teaching Hospital, Libya: Internal Medicine Resident at Brega General Hospital BGH, Libya: Faculty of Medicine, Omar Al-Mukhtar University, Al-Bayda, Libya: Faculty of Medicine of Sabha University, Sabha, Libya: Benghazi Medical Center, Benghazi, Libya: Internal Medicine Department, University of Science and Technology Hospital, Yemen: Faculty of Medicine and Health Sciences, University of Science and Technology, Yemen: Faculty of Medicine and Health Science, University of Gadarif, Sudan: Faculty of Medicine, Gabir Ibin Hayyan Medical University, Iraq: Faculty of Medicine, University of Baghdad, Iraq: College of Medicine, Al-Nahrain University, Baghdad, Iraq: Alnoor University College, Mosul, Iraq: University of Mosul, Aljumhori Hospital, Iraq: School of Medicine, University of Jordan, Amman, Jordan: Faculty of Engineering and IT, British University in Dubai, United Arab Emirates: Albalqa Applied University, Jordan: Ribat University Hospital, National Ribat University, Khartoum, Sudan: Alsalam Health Medical Hospital, Saudi Arabia; ^1^Nanotechnology Program, The American University of Cairo, Cairo, Egypt; ^2^Medical Agency for Research and Statistics, Cairo, Egypt; ^3^Department of International Business and Export Management, IMC University of Applied Management Sciences Krems, Krems an der Donau, Austria; ^4^Department of Health Sciences, St. Pölten University of Applied Sciences, St. Pölten, Austria; ^5^Faculty of Pharmacy, Beni-Suef University, Beni Suef, Egypt; ^6^Eltewacy Arab Research Group, Cairo, Egypt; ^7^Clinical Research Key, Nairobi, Kenya

**Keywords:** COVID-19, psychology, healthcare worker (HCW), depression, anxiety, stress, wellbeing

## Abstract

**Introduction:**

COVID-19, is one of the biggest challenges facing humanity in the 21st century (1). The pandemic outbreak as affected all human activities, starting with healthcare and medical service passing with economy and social relationships, as well as political, religious and cultural enactments (2). The healthcare workers were the most affected fighting in the frontline working longer hours under a high risk of being infected (3). This study aims to assess the depression, anxiety and stress levels of the healthcare workforce (physicians, nurses, pharmacists and dentists) in the Middle East and North Africa—MENA–region.

**Methods:**

We invited healthcare workers in the Middle East to participate in our cross-sectional survey by answering to the DASS-21 questionnaire.

**Results:**

A total of 4,845 healthcare workers participated in the study. The participants were from 11 countries as follows: 436 from Egypt, 430 from Algeria, 458 from Iraq, 453 from Jordan, 473 from Libya, 428 from Palestine, 419 from Saudi Arabia, 452 from Sudan, 451 from Syria, 424 from Tunisia, and 421 from Yemen. The doctors among the healthcare workers were 51.7%, 19.0% were from the nursing staff, 16.8% were pharmacists, and 12.5% were from dentists. The depression level among the healthcare workers was as follows: 29.1% were normal, 13.7% were mildly depressed, 26.9% were moderately depressed, 14.4% were suffering from severe depression, and the depression state was extremely severe for the last 15.9%. At the same time, 29.1% were suffering from no anxiety, while 6.9% were at a mild level, 22.3% were at a moderate level, 13.4% were at a severe level, and 28.3% were at an extremely severe level. For the stress levels, 38.6% were normal, 14.9% were suffering from mild stress, 20.3% were moderate, 17.4% were severe, and the stress level was extremely severe for the other 8.9%.

**Discussion:**

This study indicates that in the Middle East and North Africa—MENA—region, the prevalence of depression, anxiety, and stress among the healthcare workforce during the COVID-19 pandemic was 70.9, 70.9, 61.4, respectively.

## Introduction

SARS corona virus-2, known as COVID-19, is one of the biggest challenges facing humanity in the 21st century (1). The pandemic outbreak as affected all human activities, starting with healthcare and medical service passing with economy and social relationships, as well as political, religious and cultural enactments (2). The healthcare workers were the most affected fighting in the frontline working longer hours under a high risk of being infected (3). They were saving lives and facing enormous physical and psychological pressure, working hard and being socially isolated from their families and friends (4).

Globally health care workers were experiencing anxiety, depression, exhaustion, Post-traumatic stress disorder (PTSD), stress and mental health issues (5, 6). This study aims to assess the depression, anxiety and stress levels of the healthcare workforce (physicians, nurses, pharmacists, and dentists) in the Middle East and North Africa—MENA—region. This region is facing poverty, deficiency on the personal protective equipment (PPE) and civil wars. According to the UN Syria is one of the countries that are undergoing “the largest humanitarian crisis since the second world war” (1, 7) and Yemen where it’s crisis was described by the UN in 2017 as the worst humanitarian crisis since 1945 (8). In addition, war, riots and terrorism complicate the situation in Libya, Palestine, South Sudan, Ethiopia, Central African Republic, Mali, Niger, Burkina Faso, and Cameroon.

Our investigation includes 11 countries from the MENA region (Egypt, Algeria, Iraq, Jordon, Libya, Palestine, Saudi Arabia, Sudan, Syria, Tunisia, and Yemen) using the depression anxiety stress scale (DASS-21). In addition, our study aimed to assess the psychological effect of COVID-19 infection on the mental health of individuals by calculating the odds ratio of the psychological state of those who were infected with COVID-19 to that of others who did not have the disease. Moreover, the study aimed to assess the effect of the vaccines on the mental health of the individuals through the same criteria.

## Methodology

### Study design and participants

We invited healthcare workers in the Middle East to participate in our cross-sectional survey between November 2021 and December 2021. We created a Google survey, and the link was used by our collaborators to the healthcare workers in the 11 countries.

We used Snowball sampling (9) using a few healthcare workers as a start who met the inclusion criteria of our study and were requested to be involved in the study. The accepted volunteers are then requested to indorse other individuals who also met the study criteria and so on.

We used social networks as social media platforms such as WhatsApp and Facebook to create primary associations, catching a growing sequence of volunteers. Sampling stopped when the required sample number had been obtained.

For example, Chaim Noy (10) detected in his snowball sampling study that there was a dependence on social capital and networking. Additionally, he gained access to travelers, whereby an easy and responsive sampling method was necessary because of the flexibility and briefness of the required individuals. Furthermore, to recruit from another movable set, males who were semiprofessional motorists in Jerusalem, but establish it to be a lower easy procedure than in the travelers’ trail, difficult by doubts above his own positionality and by wrong prospects within those communicated about what the research could accomplish.

They also field visited healthcare facilities (hospitals and pharmacies) in some countries that are suffering from internet disconnection in many areas due to wars and conflicts, such as Yemen, Sudan, Syria, Libya, and Palestine.

The inclusion criteria of the investigation included (1) physicians, nurses, pharmacists, and dentists from any of the 11 countries, (2) those who were introducing healthcare at any of the healthcare facilities, and (3) 18 years old or older. Participants who reported not being from the four categories or not working in one of the healthcare facilities were excluded from the study.

### Study survey

We designed the questionnaire in two languages (Arabic and English), and it was composed of three sections, as follows:

Section 1 included the language and a brief description of the aim of the study.

Section 2 included the socio-demographic and occupational feature questions, including age (discreet between 18 and 99), gender (male or female), country (Egypt, Jordan, UAE, KSA, Yemen, Syria, Palestine, Algeria, Libya, Tunisia, Iraq, Sudan, Nigeria, and Lebanon), place of living (village, city, coastal village and desert village), work hours per week (discrete value), specialization (medical doctor, dentist, pharmacist and nursing), and type of working facility (isolation hospital, normal hospital, clinic, private clinic and pharmacy).

Section 3 included questions from the DASS-21 questionnaire. This questionnaire is a short version (21 items) of a 42-item self-report instrument designed to measure three related negative emotional states: depression, anxiety and tension/stress. It started with a brief description of the aim of the questions and how every question would be assessed and evaluated. The DASS-21 is composed of 21 questions to assess 3 different psychological distresses:

• Seven questions were used to assess the depression level by evaluating hopelessness dysphoria, self-deprecation, devaluation of life, lack of interest and involvement, anhedonia and inertia.
• Seven questions assessed anxiety levels through the assessment of autonomic arousal, skeletal musculature effect, situational anxiety, and subjective experience of anxious affect.
• Seven questions measured the level of stress by assessing difficulty relaxing, nervous arousal, easily upset agitated, irritable over reaction and impatience.

The participant must determine to what degree the question fits with and describe his feelings by choosing a number from a rating scale between 0 and 3, as score (0) means did not apply to me at all, score (1) Applied to me to some degree or some of the time, Score (2) Applied to me to a considerable degree, (3) Applied to me very much or most of the time (11).

The same section contained two more questions: the first question asks if the participant is fully vaccinated, and the second question asks if the participant has been diagnosed with COVID-19 to determine if there is any relationship between those two variables and the psychological state of the participant. All the questions were mandatory.

### Statistical analysis

The scores of every participant were calculated and multiplied by two, and then the final scores were classified as follows in Table 1.

**TABLE 1:** The psychological scale of depression anxiety stress scale (DASS-21).

Level	Depression	Anxiety	Stress
Normal	0–9	0–7	0–14
Mild	10–13	8–9	15–18
Moderate	14–20	10–14	19–25
Severe	21–27	15–19	26–33
Extremely severe	28+	20+	34+

### Ethical consideration

We obtained 10 ethical approvals from the 11 countries, one for each through our collaborators teams. The approvals were obtained from the universities, and each participant was informed that his answers would be used for research purposes without revealing his identity or his personal data, which is considered to be approval on the participant level. Furthermore, we followed all the guidelines for Good Clinical Practice and Declaration of Helsinki.

Non-linear regression models were performed using the data to assess the relationship between each of the psychological disorders and the other variables depending on the *p*-value and the confidence interval (CI). The variables that were included in each model were age, gender, work hours per week, previous COVID-19 infection, vaccination statement, number of workplaces and specialization. The data were analyzed using R software version 4.0.5 (2021-03-31), platform x86_64-w64-mingw32.

## Results

A total of 4,845 healthcare workers participated in the study. The participants were from 11 countries as follows: 436 from Egypt, 430 from Algeria, 458 from Iraq, 453 from Jordan, 473 from Libya, 428 from Palestine, 419 from Saudi Arabia, 452 from Sudan, 451 from Syria, 424 from Tunisia, and 421 from Yemen. The doctors among the healthcare workers were 51.7%, 19.0% were from the nursing staff, 16.8% were pharmacists, and 12.5% were from dentists.

The distribution of the age groups was as follows: ≤30 [2,799 (75.8)], 31–40 [1,323 (27.3)], >40 [723 (14.9)], and they were 42.5% males and 57.5 females, 50.3% reported working 40 h per week or less, 34.4% between 41 and 60 h, 11.1% between 61 and 80 h, while 4.2% only reported working for more than 80 h per week. For the living zone, 74.8 were living in a city, 10.6 were living in a coastal village, 3.2 were living in a desert village, and 11.4 were living in a village. Until the date of the end of the data collection, 68.6% reported being fully vaccinated, while 31.4% reported they were not yet fully vaccinated.

The distribution of every variable per country is reported in Table 2.

**TABLE 2:** The socio-demographic and occupational characteristics of the healthcare workers in the Middle East and North Africa (MENA) region.

			Country										
**Characteristics**		**MENA region**	**Algeria**	**Egypt**	**Iraq**	**Jordan**	**Libya**	**Palestine**	**KSA**	**Sudan**	**Syria**	**Tunisia**	**Yemen**
No. of participants		4,845	430	436	458	453	473	428	–	419	451	424	421
Age groups	≤30	2,799 (75.8)	208 (48.4)	304 (69.7)	312 (68.1)	166 (36.6)	241 (51.0)	287 (67.1)	135 (32.2)	296 (65.5)	378 (33.8)	176 (41.5)	296 (70.3)
	31–40	1,323 (27.3)	118 (27.4)	84 (19.3)	82 (17.9)	129 (28.5)	185 (39.1)	93 (21.7)	222 (53.0)	53 (11.7)	36 (8.0)	180 (42.5)	90 (21.4)
	>40	723 (14.9)	104 (24.2)	48 (11.0)	64 (14.0)	158 (34.9)	47 (9.9)	48 (11.2)	62 (14.8)	103 (22.8)	37 (8.2)	68 (16.0)	35 (8.3)
Gender	Male	2,058 (42.5)	198 (46.0)	190 (43.6)	184 (40.2)	264 (58.3)	180 (38.1)	160 (37.4)	112 (26.7)	229 (50.7)	163 (36.1)	129 (30.4)	249 (59.1)
	Female	2,787 (57.5)	232 (54.0)	246 (56.4)	274 (59.8)	189 (41.7)	293 (61.9)	268 (62.6)	307 (73.3)	223 (49.3)	288 (63.9)	295 (69.6)	172 (40.9)
Work hours per week	≤40	2,438 (50.3)	266 (61.9)	251 (57.6)	269 (58.7)	119 (26.3)	284 (60.0)	211 (49.3)	136 (32.5)	180 (39.8)	163 (36.1)	299 (70.5)	260 (61.8)
	41–60	1,667 (34.4)	158 (36.7)	146 (33.5)	144 (31.4)	180 (39.7)	131 (27.7)	174 (40.7)	260 (62.1)	145 (32.1)	106 (23.5)	104 (24.5)	119 (28.3)
	61–80	537 (11.1)	6 (1.4)	31 (7.1)	31 (6.8)	149 (32.9)	53 (11.2)	32 (7.5)	21 (5.0)	74 (16.4)	89 (19.7)	20 (4.7)	31 (7.4)
	>80	203 (4.2)	–	8 (1.8)	14 (3.1)	5 (1.1)	5 (1.1)	11 (2.6)	2 (0.5)	53 (11.7)	93 (20.6)	1 (0.2)	11 (2.6)
Living zone	City	3,624 (74.8)	202 (47.0)	233 (53.4)	425 (92.8)	375 (82.8)	286 (60.5)	286 (66.8)	353 (84.2)	391 (86.5)	408 (90.5)	268 (63.2)	397 (94.3)
	Coastal village	515 (10.6)	123 (28.6)	60 (13.8)	1 (0.2)	29 (6.4)	100 (21.1)	12 (2.8)	28 (6.7)	7 (1.5)	12 (2.7)	136 (32.1)	7 (1.7)
	Desert village	156 (3.2)	69 (16.0)	7 (1.6)	1 (0.2)	16 (3.5)	39 (8.2)	3 (0.7)	12 (2.9)	2 (0.4)	1 (0.2)	6 (1.4)	–
	Village	550 (11.4)	36 (8.4)	136 (31.2)	31 (6.8)	33 (7.3)	48 (10.1)	127 (29.7)	26 (6.2)	52 (11.5)	30 (6.7)	14 (3.3)	17 (4.0)
Occupation	Doctor	2,506 (51.7)	188 (43.7)	96 (22.0)	212 (46.3)	238 (52.5)	291 (61.5)	146 (34.1)	259 (61.8)	305 (67.5)	283 (62.7)	257 (60.6)	231 (54.9)
	Nursing	922 (19.0)	59 (13.7)	55 (12.6)	71 (15.5)	65 (14.3)	49 (10.4)	148 (34.6)	116 (27.7)	49 (10.8)	133 (29.5)	114 (26.9)	63 (15.0)
	Pharmacist	813 (16.8)	78 (18.1)	251 (57.6)	94 (20.5)	55 (12.1)	79 (16.7)	60 (14.0)	28 (6.7)	63 (13.9)	25 (5.5)	25 (5.9)	55 (13.1)
	Dentist	604 (12.5)	105 (24.4)	34 (7.8)	81 (17.7)	95 (21.0)	54 (11.4)	74 (17.3)	16 (3.8)	35 (7.7)	10 (2.2)	28 (6.6)	72 (17.1)
Ex-Covid-19 inf.	Yes	1,971 (40.7)	251 (58.4)	177 (40.6)	256 (55.9)	158 (34.9)	178 (37.6)	213 (49.8)	142 (33.9)	140 (31.0)	168 (37.3)	192 (45.3)	96 (22.8)
	No	2,874 (59.3)	179 (41.6)	259 (59.4)	202 (44.1)	295 (65.1)	295 (62.4)	215 (50.2)	277 (66.1)	312 (69.0)	283 (62.7)	232 (54.7)	325 (77.2)
If fully vaccinated	Yes	3,324 (68.6)	275 (64.0)	324 (74.3)	400 (87.3)	416 (91.8)	244 (51.6)	386 (90.2)	406 (96.9)	311 (68.8)	146 (32.4)	373 (88.0)	43 (10.2)
	No	1,521 (31.4)	155 (36.0)	112 (25.7)	58 (12.7)	37 (8.2)	229 (48.4)	42 (9.8)	13 (3.1)	141 (31.2)	305 (67.6)	51 (12.0)	378 (89.8)

A. Socio-demographic
B. Psychological state

The depression level among the healthcare workers was as follows: 29.1% were normal, 13.7% were mildly depressed, 26.9% were moderately depressed, 14.4% were suffering from severe depression, and the depression state was extremely severe for the last 15.9%. At the same time, 29.1% were suffering from no anxiety, while 6.9% were at a mild level, 22.3% were at a moderate level, 13.4% were at a severe level, and 28.3% were at an extremely severe level. For the stress levels, 38.6% were normal, 14.9% were suffering from mild stress, 20.3% were moderate, 17.4% were severe, and the stress level was extremely severe for the other 8.9%.

The psychological state per occupation showed some variance between the different occupations, and it is reported in Table 3.

**TABLE 3:** Psychological state per occupation.

		MENA region	Occupation			
			**Doctors**	**Nursing**	**Pharmacists**	**Dentists**
The psychological disorder	The level	4,845	2,506 (51.7)	922 (19.0)	813 (16.8)	604 (12.5)
Depression	Normal	1,409 (29.1)	766 (30.6)	282 (30.6)	216 (26.6)	145 (24.0)
	Mild	663 (13.7)	338 (13.5)	127 (13.8)	118 (14.5)	80 (13.2)
	Moderate	1,302 (26.9)	639 (25.5)	242 (26.2)	216 (26.6)	205 (33.9)
	Severe	699 (14.4)	359 (14.3)	135 (14.6)	118 (14.5)	87 (14.4)
	Extremely severe	772 (15.9)	404 (16.1)	136 (14.8)	145 (17.8)	87 (14.4)
Anxiety	Normal	1,410 (29.1)	800 (31.9)	274 (29.7)	202 (24.8)	134 (22.2)
	Mild	334 (6.9)	188 (7.5)	49 (5.3)	61 (7.5)	36 (6.0)
	moderate	1,080 (22.3)	546 (21.8)	196 (21.3)	180 (22.1)	158 (26.2)
	Severe	648 (13.4)	322 (12.8)	120 (13.0)	104 (12.8)	102 (16.9)
	Extremely severe	1,373 (28.3)	650 (25.9)	283 (30.7)	266 (32.7)	174 (28.8)
Stress	Normal	1,870 (38.6)	936 (37.4)	400 (43.4)	293 (36.0)	241 (39.9)
	Mild	720 (14.9)	367 (14.6)	116 (12.6)	136 (16.7)	101 (16.7)
	Moderate	983 (20.3)	532 (21.2)	181 (19.6)	157 (19.3)	113 (18.7)
	Severe	843 (17.4)	438 (17.5)	154 (16.7)	155 (19.1)	96 (15.9)
	Extremely severe	429 (8.9)	233 (9.3)	71 (7.7)	72 (8.9)	53 (8.8)

**FIGURE 1:**
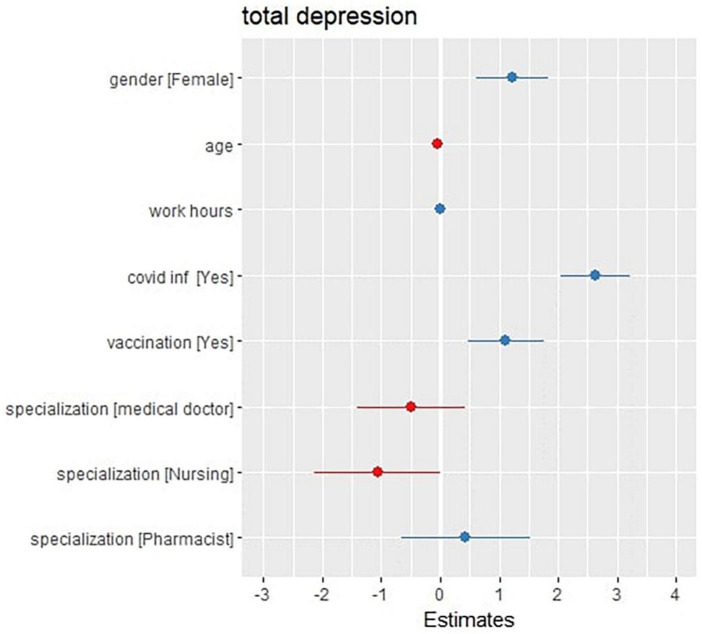
Forest (dot whisker) plot of the depression generalized non-linear regression model (shows the positions and direction of significance).

As shown in Figure 1, the first regression generalized non-linear model is used to assess whether there is any type of association between the total depression score and the other variables, such as age, gender, work hours per week, previous COVID infection, vaccination state, and occupation. The model represents a residual deviance value of 515,222 on 4,842 degrees of freedom.

The model shows that some variables, such as being an ex-COVID-19 patient (at a CI 2.04–3.22, *P*-value = 0.001 and a total increase by 2.36), being fully vaccinated (at a CI 0.48–1.75, *P*-value = 0.001 and a total increase by 1.12), and being a female (at a CI 0.61–1.83, *P*-value < 0.001 and a total increase by 1.22), are significantly associated with the elevation of the total depression score, while other factors, such as getting old (at a CI –0.07 to 0.00, *P*-value = 0.027 and a total decrease by 0.04), and being male (compared to the females), are significantly associated with a decrease in the total depression score (as shown in Figure 1). Despite being non-significant, there was also an association with occupation as a medical doctor (with a total decrease of 0.49) and as a nurse (with a total decrease of 1.06) with a decrease in the total depression score. Additionally, the model showed no significant association with working hours.

**FIGURE 2:**
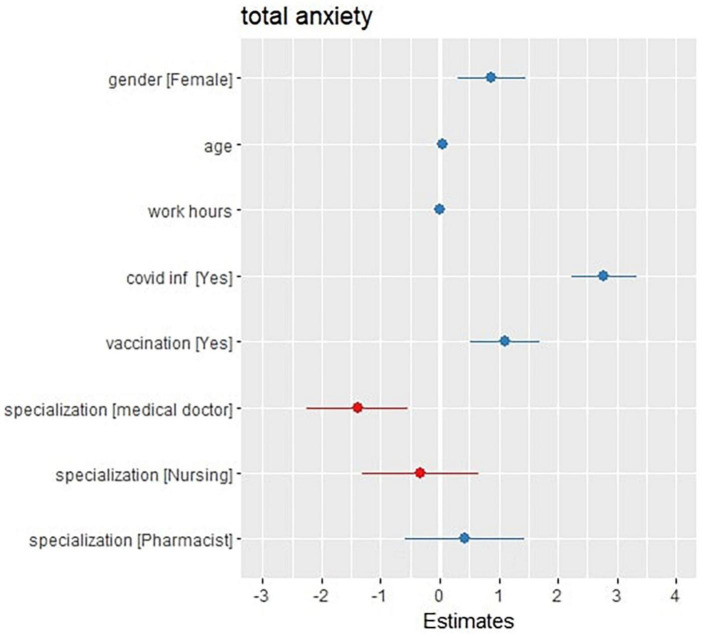
Forest (dot whisker) plot of the anxiety generalized non-linear regression model (shows the positions and direction of significance).

As shown in Figure 2, the second regression model investigated any association between the total anxiety score and the (age, gender, work hours per week, previous COVID-19 infection, vaccination state and occupation) variables. The model represents a residual deviance of 444,877 on 4,842 degrees of freedom.

The model shows that getting old (at CI 0.01–0.08, *P*-value = 0.004 and a total increase by 0.04), being an ex-COVID-19 patient (at CI 2.23–3.33, *P*-value < 0.001 and a total increase by 2.78), being fully vaccinated (at a CI 0.52 – 1.70 and a total increase by 1.11), and being a female (at a CI 0.32–1.45, *P*-value 0.002 and a total increase by 0.88) are significantly associated variables with the elevation of the total anxiety scores of the participants, while working as a doctor is significantly associated with the decrease in the total anxiety scores at a CI of –0.25 to –0.54, *P*-value = 0.001 and a total decrease of 1.40. The model also shows that some variables, such as the work hours per week and occupation as a pharmacist or a nurse compared with having a dentist, have no significant association with the total anxiety scores of the participants.

As shown in Table 4, the last generalized non-linear regression model investigated any association between the total stress score for the participants and the other variables (age, sex, work hours per week, previous COVID-19 infection, vaccination state and occupation). The model represents a residual deviance of 498,266 on 4,842 degrees of freedom.

**TABLE 4:** The prevalence of depression, anxiety and stress levels among the healthcare workers in the MENA region.

The psychological disorder	The level	MENA region	Country										
			**Algeria**	**Egypt**	**Iraq**	**Jordan**	**Libya**	**Palestine**	**KSA**	**Sudan**	**Syria**	**Tunisia**	**Yemen**
Depression	Normal	1,409 (29.1)	51 (11.9)	103 (23.6)	51 (11.1)	89 (19.6)	187 (39.5)	126 (29.4)	191 (45.6)	157 (34.7)	134 (29.7)	121 (28.5)	199 (47.3)
	Mild	663 (13.7)	37 (8.6)	61 (14.0)	58 (12.7)	76 (16.8)	59 (12.5)	62 (14.5)	47 (11.2)	68 (15.0)	86 (19.1)	44 (10.4)	65 (15.4)
	Moderate	1,302 (26.9)	174 (40.5)	110 (25.2)	137 (29.9)	172 (38.0)	88 (18.6)	141 (32.9)	87 (20.8)	89 (19.7)	120 (26.6)	110 (25.9)	199 (47.3)
	severe	699 (14.4)	80 (18.6)	68 (15.6)	96 (21.0)	73 (16.1)	79 (16.7)	53 (12.4)	35 (8.4)	51 (11.3)	66 (14.6)	61 (14.4)	37 (8.8)
	Extremely severe	772 (15.9)	88 (20.5)	94 (21.6)	116 (25.3)	43 (9.5)	60 (12.7)	46 (10.7)	59 (14.1)	87 (19.2)	45 (10.0)	88 (20.8)	46 (10.9)
Anxiety	Normal	1,410 (29.1)	60 (14.0)	105 (12.4)	75 (16.4)	65 (14.3)	170 (35.9)	142 (33.2)	178 (42.5)	131 (29.0)	173 (38.4)	139 (32.8)	172 (40.9)
	Mild	334 (6.9)	35 (8.1)	30 (6.9)	27 (5.9)	18 (4.0)	28 (5.9)	34 (7.9)	23 (5.5)	39 (8.6)	30 (6.7)	32 (7.5)	38 (9.0)
	moderate	1,080 (22.3)	150 (34.9)	91 (20.9)	84 (18.3)	98 (21.6)	94 (19.9)	102 (23.8)	68 (16.2)	120 (26.5)	110 (24.4)	78 (18.4)	85 (20.2)
	Severe	648 (13.4)	103 (24.0)	54 (12.4)	68 (14.8)	92 (20.3)	45 (9.5)	42 (9.8)	42 (10.0)	57 (12.6)	56 (12.4)	39 (9.2)	50 (11.9)
	Extremely severe	1,373 (28.3)	82 (19.1)	156 (35.8)	204 (44.5)	180 (39.7)	136 (28.8)	108 (25.2)	108 (25.8)	105 (23.2)	82 (18.2)	136 (32.1)	76 (18.1)
Stress	Normal	1,870 (38.6)	124 (28.8)	105 (24.1)	75 (16.4)	65 (14.3)	170 (35.9)	142 (33.2)	178 (42.5)	131 (29.0)	173 (38.4)	139 (32.8)	172 (40.9)
	Mild	720 (14.9)	91 (21.2)	30 (6.9)	27 (5.9)	18 (4.0)	28 (5.9)	34 (7.9)	23 (5.5)	39 (8.6)	30 (6.7)	32 (7.5)	38 (9.0)
	Moderate	983 (20.3)	91 (21.2)	91 (20.9)	84 (18.3)	98 (21.6)	94 (19.9)	102 (23.8)	68 (16.2)	120 (26.5)	110 (24.4)	78 (18.4)	85 (20.2)
	Severe	843 (17.4)	88 (20.5)	54 (12.4)	68 (14.8)	92 (20.3)	45 (9.5)	42 (9.8)	42 (10.0)	57 (12.6)	56 (12.4)	39 (9.2)	50 (11.9)
	Extremely severe	429 (8.9)	36 (8.4)	156 (35.8)	204 (44.5)	180 (39.7)	136 (28.8)	108 (25.2)	108 (25.8)	105 (23.2)	82 (18.2)	136 (32.1)	76 (18.1)

**FIGURE 3:**
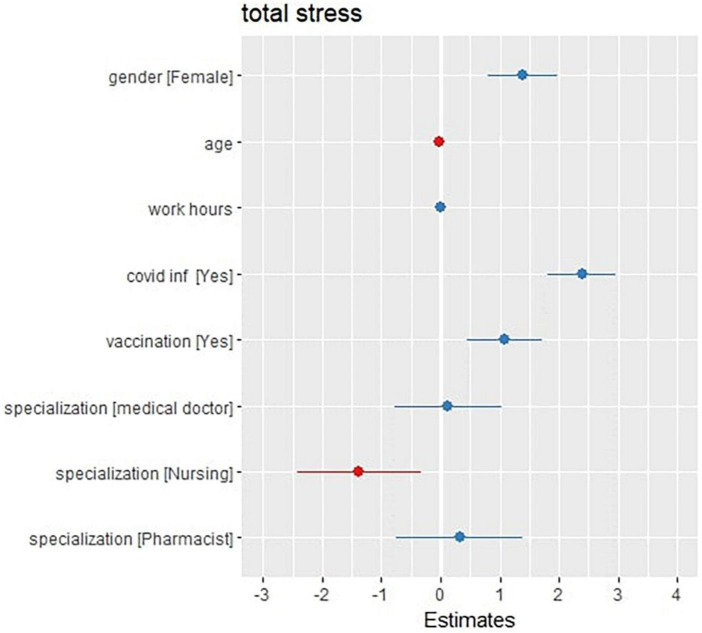
Forest (dot whisker) plot of the stress generalized non-linear regression model (shows the positions and direction of significance).

As shown in Figure 3, the third regression model investigated any association between the total stress score and the (age, gender, work hours per week, previous COVID-19 infection, vaccination state and occupation) variables. The model represents a residual deviance of 444,877 on 4,842 degrees of freedom.

The model shows that some variables, such as being an ex-COVID-19 patient (at a CI 1.80–2.97, *P*-value < 0.001 and a total increase by 2.39), being fully vaccinated (at a CI 0.45–1.71, *P*-value 0.001 and a total increase by 1.08), and being a female (at a CI 0.79–1.98, *P*-value < 0.001, and a total increase by 1.39), are significantly associated with the elevation of the total stress scores of the participants, while other variables, such as being male (compared with the females) and being a nurse (at a CI –2.42 to –0.33, *P*-value = 0.01 and a total decrease by 1.38), are associated with the decrease of the total stress score of the participants. The model also showed that some other variables, such as age, the work hours per week, and the occupation as a doctor or pharmacist, have no association with the total stress scores that are recorded to the participants.

## Discussion

This study indicates that in the Middle East and North Africa—MENA—region, the prevalence of depression, anxiety, and stress among the healthcare workforce during the COVID-19 pandemic was 70.9, 70.9, 61.4, respectively. Similarly, the levels in some countries, such as Egypt and Saudi Arabia, were previously investigated in another study by Arafa et al. (12) which found that 69% of the healthcare workers in both countries were depressed, 58.9% had anxiety, and 55.9% had stress. In contrast, another study in Singapore in 2020 during the COVID-19 pandemic reported a prevalence of depression of 8.1%, anxiety of 10.8% and stress of 6.4%, which are significantly lower than those in the MENA region. The prevalence of depression levels was 42.8% normal/mild and 57.2% moderate to extremely severe. The prevalence of anxiety levels was 36% normal/mild and 64% moderate to extremely severe. The study also showed the prevalence of stress levels among the healthcare workforce to be 53.4% normal/mild and 46.6% moderate to extremely severe. The percentage of those with mild to extremely severe depression, stress or anxiety in the MENA region is higher than that in European countries. Hummel et al. (13) represented depression levels in eight European countries as 30.51% moderate to extremely severe, anxiety levels as 32.2% moderate to extremely severe and stress levels as 41.24% moderate to extremely severe.

In the study of Chen et al. (20) it was found that the pooled prevalence rate for anxiety in the African population is 37%, which is significantly higher than those in China reported in Bareeqa et al. (14) (22%; *p*-value < 0.0001) and Pappa et al. (5) (23%; *p*-value < 0.0001). However, no, the pooled prevalence rate for anxiety in South Asian countries is significantly higher than that in Africa (41.3%; *p*-value < 0.0001) (15). In addition, the pooled prevalence of anxiety in Africa (37%) is higher than those in individual cross-country individual studies, such as a study of 10 countries (China, India, Japan, Iran, Iraq, Italy, Nepal, Nigeria, Spain, and the UK) (32%; *p*-value < 0.0001) (16) and a study in 17 countries in the regions of Asia (China, Pakistan, India, Japan, Singapore, Vietnam), Latin America (Argentina, Brazil, Chile, and Mexico) (33%; *p*-value < 0.0001) and middle East (Palestine, Iran), Europe (Denmark, Greece, Turkey, Italy, Spain) (17). Moreover, they find that the pooled prevalence rate of anxiety among frontline HCWs in Africa (51%) is significantly higher than Bareeqa et al. (14) (24%; *p*-value < 0.0001), Krishnamoorthy et al. (19) (26%; *p*-value < 0.0001), and Ren et al. (18) (27%; *p*-value < 0.01). Similarly, we find that the pooled prevalence rate of anxiety among the general population (37%) in Africa is significantly higher than Ren et al. (18) (24%; *p*-value < 0.0001). The pooled prevalence rate for depression in the African population (45%) is significantly higher than those in China reported by Bareeqa et al. (14) (27%; *p*-value < 0.0001), Pappa et al. (5) (23%; *p*-value < 0.0001), Krishnamoorthy et al. (19) (26%; *p*-value < 0.0001), and Ren et al. (18) (28%; *p*-value < 0.0001). The pooled prevalence rate for depression in the African population (45%) is higher than those in Spain (23%; *p*-value < 0.0001) (20). In South Asian countries reported by Hossain et al. (15) (34%; *p*-value < 0.0001). The pooled prevalence for depression in Africa (45%) is also higher than the pooled prevalence in a study including over 17 countries reported by Luo et al. (17) (28%; *p*-value < 0.01) and another study of 10 countries reported by Salari et al. (16) (34%; *p*-value < 0.01). However, the prevalence rate for depression in Africa is lower than Italy which is the country with the highest prevalence for depression (67%) (17). In addition, the pooled prevalence rates of anxiety and depression in Sub-Saharan Africa were found to be (31 and 30%) which are lower than those reported in North Africa (44 and 55%) that may suggest that there is a high heterogeneous prevalence of mental health symptoms within the regions of Africa, and this heterogeneous prevalence rate between Sub-Saharan Africa and North Africa may arise because of the lack of awareness of the danger of COVID-19 as a result of the insufficient COVID-19 testing or the lower death rates due to the younger population in Sub-Saharan Africa (21).

To assess the mental health symptoms in southeast Asia, 32 samples from 25 studies including 20,352 persons were included in the study of Pappa et al. (22). Depression in 15 studies and anxiety was assessed in 25 studies and the prevalence rates were 16 and 22%, respectively. In addition, the prevalence of anxiety and depression was similar among general HCWs (17%), frontline HCWs (18%) while being higher in the general population (27%), which is lower than the recorded scores of 33 and 32% for anxiety and 28 and 34% for depression in the meta-analysis by Luo et al. (17) from 17 countries (China, Singapore, India, Japan, Pakistan, Vietnam, Iran, Palestine, Italy, Spain, Turkey, Denmark Greece, Argentina, Brazil, Chile, and Mexico), and the meta-analysis by Salari et al. (23) from 10 countries (China, India, Japan, Iran, Iraq, Italy, Nepal, Nigeria, Spain, and UK), Respectively.

Our study showed a significant association between the total DASS-21 scores of the participants and the other variables. Regarding age, getting old is associated with dissension in the depression total score and elevation of the anxiety total score. Regarding sex, the three models showed that males had dissension in the total scores of depression, anxiety and stress by 1.27, 0.92, and 1.44, respectively, compared with females. Regarding occupation, doctors had dissension in the total scores of depression and anxiety, and nurses had dissension in the total scores of depression and stress. Additionally, being an ex-COVID-19 patient showed a significant positive association with depression, anxiety, and stress. Our findings of depression, anxiety, and stress risk factors differ from those of other studies. For example, a cross-sectional survey in 12 Arab countries reported that the prevalence of mental health symptoms was higher in healthcare workers (HCWs) aged 30–39 years, those who worked > 44 h/week, those in contact with COVID-19 cases, and HCWs who were not satisfied with the preventive measures. The prevalence of mental health symptoms was lower among male HCWs (14).

In addition, a multiple-method design in the Bangkok study determined the risk factors for emotional exhaustion, which were male sex, nurses, doctors, working in the COVID-19 inpatient unit, and working in the COVID-19 intensive care unit. Additionally, pre-existing mental illness was associated with anxiety, depression, and PTSD (5). Furthermore, In U.S, a cross-sectional survey found that higher levels of anxiety were observed with younger ages and female gender, while occupational roles with increased exposure risk did not report higher levels of anxiety (15). Moreover, being fully vaccinated was significantly associated with elevated depression, anxiety, and stress total scores. The results could be considered evidence of the association between the psychiatric statement of the participants and the vaccines. A cross-sectional study in Iraq illustrated that the majority of participants had a normal level of DASS-21 after receiving the vaccine (80% were females). Higher scores were obtained among graduated young age groups and among individuals who had side effects associated with the vaccine (16). Another investigation by Perez-Arce F et al. (17) reported a decrease in mental distress after receiving the first dose of the COVID-19 vaccine.

## Conclusion

COVID-19 had critical psychological effects on the medical health workforce in the Middle East and North Africa—MENA—region. Being women, being ex-COVID-19 and being fully vaccinated were associated with an increased risk of depression, anxiety and stress. They required special attention, health-related education, and psychological support. Additionally, to improve their mental health, strategies such as supportive and respectful colleagues, appropriate financial compensation, reduced workload, clarity of policy and communication channels, and adequate personal protective equipment should be implemented.

## Limitations

The study has some limitations. First, snowball sampling was used. This may cause oversampling a particular network of peers to lead to bias. Additionally, respondents may be hesitant to provide names of peers, and asking them to do so may raise ethical concerns. Furthermore, there is no guarantee about the representativeness of the samples. In addition, it is not possible to determine the actual pattern of distribution of the population. Moreover, it is not possible to determine the sampling error and make statistical inferences from the sample to the population due to the absence of random selection of samples. Finally, the age group >40 is relatively underrepresented in comparison to the other age groups, which also leads to bias.

## Data availability statement

The original contributions presented in this study are included in this article/supplementary material, further inquiries can be directed to the corresponding author.

## Ethics statement

The studies involving human participants were reviewed and approved by Institutional Review Board Committee (IRB) in Egypt, Palestine, Yemen, Iraq, Libya, Sudan, Syria, Tunisia, Saudi, Jordan. The patients/participants were informed that their answers will be used in this study.

## The EARG group (Eltewacy Arab research group)

DMD, Algiers Department of Dental Medicine, Algeria: Mohammed Amir Rais. Faculty of Medicine, Al-Azhar University, Egypt: Reman Ashraf. Faculty of Pharmacy, Damanhour University, Egypt: Entesar kamal abdou. Faculty of Pharmacy, Sohag University, Sohag, Egypt: Ereen Sobhy Kiddees and Arwa Abdelrazek Abdellah. Faculty of Pharmacy (Boys), Al-Azhar University, Assiut Branch, Egypt: Mostafa S. M. Abd El-Maksoud. Faculty of Pharmacy Mansoura University, Egypt: Aseel MitwallyAwad. Faculty of Medicine and Health Sciences, An-Najah National University, Nablus, Palestine: Ahmad Hanani, Duha Yousef Suboh, Samaa Elkhader, and Salsabeel Hanayshe. Faculty of Medicine, Al-Quds University, Palestine: Gharam Kiswani. Faculty of Medicine of Tunis, University Tunis El Manar, Tunisia: Hanen Ben Ammar and Ghada Hamdi. Faculty of Medicine of Monastir, Tunisia: Lina Brahmi l. Faculty of Medicine Sfax, Sfax University Tunisia, Sfax, Tunisia: Malek mohamed Ayadi. Faculty of Medicine, Aleppo University, Syria: Bayan Zitani, Nour Karkar, Tasneem othman, Tasnim Assalyeh, Batoul Al Sari, and Baraa Altweel. College of Medical Technology, Department of Public Health, Derna, Libya: Raga A. Elzahaf. Radiologist Almogarief Teaching Hospital, Libya: Moufiq abdulrasoul Hasan. Internal Medicine Resident at Brega General Hospital BGH, Libya: Almajdoub Ali Mohammed. Faculty of Medicine, Omar Al-Mukhtar University, Al-Bayda, Libya: Wisam A. Hasan. Faculty of Medicine of Sabha University, Sabha, Libya: Hajar Alkokhiya Aldare. Benghazi Medical Center, Benghazi, Libya: Ayoub Akwaisah. Internal Medicine Department, University of Science and Technology Hospital, Yemen: Ali Ahmed Awas, Dares Fateh Mohammed, and Hareth Al-duais. Faculty of Medicine and Health Sciences, University of Science and Technology, Yemen: Kholood Ahmed Al-subari and Badr Alhalool. Faculty of Medicine and Health Science, University of Gadarif, Sudan: Ahmed Abdelrahim Musbah, Elhusseini Abdelrahim Musbah, Hassan Salih Eisa, Khider Altayeb Bakheet, and Mohammed Al Monje Amir. Faculty of Medicine, Gabir Ibin Hayyan Medical University, Iraq: Ali Riyadh abdulabbas. Faculty of Medicine, University of Baghdad, Iraq: Jaafar D. Al-Dabagh. College of Medicine, Al-Nahrain University, Baghdad, Iraq: Karrar H. Alnajjar. Alnoor University College, Mosul, Iraq: Mareb Hameed Ahmed. University of Mosul, Aljumhori Hospital, Iraq: Daniah Adil Tawfeeq. School of Medicine, University of Jordan, Amman, Jordan: Wahid Aloweiwi and Zaid Alkayed. Faculty of Engineering and IT, British University in Dubai, United Arab Emirates: Abdullah Sughayer. Albalqa Applied University, Jordan: Aya Thaer Albaddawe and Lina A. Abu-Sirhan. Ribat University Hospital, National Ribat University, Khartoum, Sudan: Hind AbdAlla Hamad and Omnia Omer Ibrahim. Alsalam Health Medical Hospital, Saudi Arabia: Asmaa abdulmonem Ahmed.

## Author contributions

ME-G: conceptualization and supervision. ME-G, TO, and AE: data curation. NE and ME-G: collecting the authors team. MR, WA, DS, MA, BZ, RE, AAw, AMu, and HH: country specific team leaders. TO, AE, and NH: data analysis and data interpretation. TO and NH: validation. WA, RA, AH, HA, BZ, AMo, AAw, AMu, AR, and HH: ethical approvals. TO and ME-G: writing—original draft. ME-G and NH: writing—review and editing. All authors approved the final draft of the manuscript.
